# Supplementary material for: Single Cell Transcriptomic and Chromatin Profiles Suggest Layer Vb Is the Only Layer With Shared Excitatory Cell Types in the Medial and Lateral Entorhinal Cortex
Source: Front Neural Circuits. 2022 Jan 26;15:806154. doi: 10.3389/fncir.2021.806154 (PMC8826650; doi:10.3389/fncir.2021.806154)
Supplement: Supplementary file 14 [file Data_Sheet_1.ZIP › 15/knownResults.html]

15 - Homer Known Motif Enrichment Results


# Homer Known Motif Enrichment Results (15)

Homer *de novo* Motif Results  
Gene Ontology Enrichment Results  
Known Motif Enrichment Results (txt file)  
Total Target Sequences = 4859, Total Background Sequences = 44428

|  |  |  |  |  |  |  |  |  |  |  |  |
| --- | --- | --- | --- | --- | --- | --- | --- | --- | --- | --- | --- |
| Rank | Motif | Name | P-value | log P-pvalue | q-value (Benjamini) | # Target Sequences with Motif | % of Targets Sequences with Motif | # Background Sequences with Motif | % of Background Sequences with Motif | Motif File | SVG |
| 1 | A C T G T C A G A G C T G A C T C A T G A G T C A G T C G C T A C G A T C T A G T C A G G T A C C T G A T C G A | Rfx1(HTH)/NPC-H3K4me1-ChIP-Seq(GSE16256)/Homer | 1e-158 | -3.638e+02 | 0.0000 | 465.0 | 9.57% | 924.1 | 2.08% | motif file (matrix) | svg |
| 2 | C T A G A G C T G A C T C A T G A G T C A G T C G T C A C A G T C T A G T C A G G T A C C T G A T C G A G A T C T G A C | Rfx2(HTH)/LoVo-RFX2-ChIP-Seq(GSE49402)/Homer | 1e-154 | -3.560e+02 | 0.0000 | 310.0 | 6.38% | 394.1 | 0.89% | motif file (matrix) | svg |
| 3 | A G T C A T C G C T A G A G C T G A C T C T A G A G T C A G T C G C T A C A G T T C A G T C A G G A T C C T G A T C G A G A T C | RFX(HTH)/K562-RFX3-ChIP-Seq(SRA012198)/Homer | 1e-148 | -3.409e+02 | 0.0000 | 281.0 | 5.78% | 331.7 | 0.75% | motif file (matrix) | svg |
| 4 | G T A C G A T C C A G T A G T C A G T C A G T C T G C A G A T C C T G A A T G C G T C A A C G T | WT1(Zf)/Kidney-WT1-ChIP-Seq(GSE90016)/Homer | 1e-147 | -3.385e+02 | 0.0000 | 672.0 | 13.83% | 1955.8 | 4.40% | motif file (matrix) | svg |
| 5 | C A G T T C A G G A T C A C T G A C G T C T A G A C T G A C T G G A C T C T A G | Egr1(Zf)/K562-Egr1-ChIP-Seq(GSE32465)/Homer | 1e-140 | -3.231e+02 | 0.0000 | 753.0 | 15.49% | 2451.5 | 5.52% | motif file (matrix) | svg |
| 6 | A C T G C T A G T C G A C G A T C A T G G C T A A T C G C G A T G T A C G C T A A G C T G T A C | Fra1(bZIP)/BT549-Fra1-ChIP-Seq(GSE46166)/Homer | 1e-139 | -3.202e+02 | 0.0000 | 919.0 | 18.91% | 3416.1 | 7.69% | motif file (matrix) | svg |
| 7 | C A T G C T A G A G C T G A C T C A T G A G T C G A T C G C T A C G A T C T A G T C A G G T A C C T G A T C G A | X-box(HTH)/NPC-H3K4me1-ChIP-Seq(GSE16256)/Homer | 1e-138 | -3.187e+02 | 0.0000 | 337.0 | 6.93% | 544.4 | 1.23% | motif file (matrix) | svg |
| 8 | C T A G T C G A G C A T C A T G G C T A T A G C C G A T G T A C C T G A A G C T | JunB(bZIP)/DendriticCells-Junb-ChIP-Seq(GSE36099)/Homer | 1e-138 | -3.182e+02 | 0.0000 | 884.0 | 18.19% | 3221.0 | 7.25% | motif file (matrix) | svg |
| 9 | C T A G T C G A A C G T A C T G C G T A A T G C A C G T G T A C C G T A A G C T G A T C G T A C | Atf3(bZIP)/GBM-ATF3-ChIP-Seq(GSE33912)/Homer | 1e-134 | -3.098e+02 | 0.0000 | 1014.0 | 20.86% | 4059.8 | 9.14% | motif file (matrix) | svg |
| 10 | C A G T T G C A A C G T A C T G C G T A A T C G C G A T T G A C C G T A A C G T | BATF(bZIP)/Th17-BATF-ChIP-Seq(GSE39756)/Homer | 1e-132 | -3.057e+02 | 0.0000 | 1011.0 | 20.80% | 4067.8 | 9.15% | motif file (matrix) | svg |
| 11 | A C T G C A T G T C G A A C G T A C T G C G T A A T C G A C G T G T A C C G T A G A C T A G T C | Fos(bZIP)/TSC-Fos-ChIP-Seq(GSE110950)/Homer | 1e-132 | -3.047e+02 | 0.0000 | 944.0 | 19.42% | 3659.9 | 8.24% | motif file (matrix) | svg |
| 12 | T C G A A C G T C A T G G C T A T A G C C G A T G T A C G C T A A C G T A T G C | AP-1(bZIP)/ThioMac-PU.1-ChIP-Seq(GSE21512)/Homer | 1e-126 | -2.911e+02 | 0.0000 | 1058.0 | 21.77% | 4464.9 | 10.05% | motif file (matrix) | svg |
| 13 | C A T G C T A G T C G A A C G T A C T G C G T A T A G C C G A T T G A C C G T A A G C T G A T C | Fra2(bZIP)/Striatum-Fra2-ChIP-Seq(GSE43429)/Homer | 1e-118 | -2.726e+02 | 0.0000 | 751.0 | 15.45% | 2700.7 | 6.08% | motif file (matrix) | svg |
| 14 | C T A G T C G A C G A T A C T G C G T A T A C G A G C T T G A C G C T A A C G T G A T C T A G C | Fosl2(bZIP)/3T3L1-Fosl2-ChIP-Seq(GSE56872)/Homer | 1e-102 | -2.350e+02 | 0.0000 | 534.0 | 10.99% | 1688.0 | 3.80% | motif file (matrix) | svg |
| 15 | C A T G A G T C G A C T C G T A C G A T G C A T G A C T G C A T C G A T C T A G C A T G T G A C | Mef2b(MADS)/HEK293-Mef2b.V5-ChIP-Seq(GSE67450)/Homer | 1e-96 | -2.217e+02 | 0.0000 | 1321.0 | 27.18% | 6862.6 | 15.44% | motif file (matrix) | svg |
| 16 | C A T G G T A C G A C T G C T A C G T A C G T A C G T A G C T A G A C T C T G A T C A G G T A C | Mef2c(MADS)/GM12878-Mef2c-ChIP-Seq(GSE32465)/Homer | 1e-89 | -2.061e+02 | 0.0000 | 882.0 | 18.15% | 3956.7 | 8.90% | motif file (matrix) | svg |
| 17 | A T G C A G T C G T A C A G C T T C G A C T A G G A T C C T G A G T C A A G T C G C T A T C A G | Rfx5(HTH)/GM12878-Rfx5-ChIP-Seq(GSE31477)/Homer | 1e-89 | -2.053e+02 | 0.0000 | 637.0 | 13.11% | 2426.7 | 5.46% | motif file (matrix) | svg |
| 18 | C T A G T C G A A C G T A C T G C G T A T A G C C G A T G T A C C G T A A G C T G A T C G T A C | Jun-AP1(bZIP)/K562-cJun-ChIP-Seq(GSE31477)/Homer | 1e-88 | -2.045e+02 | 0.0000 | 415.0 | 8.54% | 1207.8 | 2.72% | motif file (matrix) | svg |
| 19 | C A G T T C A G A G C T G A C T A C G T A G T C G A T C G A C T C T G A A C T G G A T C C G T A C T G A A G T C G T A C | Rfx6(HTH)/Min6b1-Rfx6.HA-ChIP-Seq(GSE62844)/Homer | 1e-58 | -1.355e+02 | 0.0000 | 1047.0 | 21.54% | 5810.7 | 13.08% | motif file (matrix) | svg |
| 20 | G T A C G A C T C G T A C T G A T C G A C G T A G C T A C A G T C T G A T A C G | Mef2a(MADS)/HL1-Mef2a.biotin-ChIP-Seq(GSE21529)/Homer | 1e-57 | -1.329e+02 | 0.0000 | 692.0 | 14.24% | 3322.7 | 7.48% | motif file (matrix) | svg |
| 21 | C A T G A G T C A G C T C G T A C G A T C G A T G C A T G C A T C G A T C T G A C A T G T G A C | Mef2d(MADS)/Retina-Mef2d-ChIP-Seq(GSE61391)/Homer | 1e-50 | -1.160e+02 | 0.0000 | 374.0 | 7.70% | 1436.4 | 3.23% | motif file (matrix) | svg |
| 22 | C G A T T A C G T G A C G A C T C A T G C G T A T A C G A C G T G T A C C T G A | Bach2(bZIP)/OCILy7-Bach2-ChIP-Seq(GSE44420)/Homer | 1e-49 | -1.128e+02 | 0.0000 | 287.0 | 5.91% | 963.9 | 2.17% | motif file (matrix) | svg |
| 23 | C T A G T C A G C A G T T C A G A C T G A C T G G A T C C T A G A C T G C T A G T C A G A T G C | KLF14(Zf)/HEK293-KLF14.GFP-ChIP-Seq(GSE58341)/Homer | 1e-48 | -1.122e+02 | 0.0000 | 1012.0 | 20.82% | 5850.0 | 13.16% | motif file (matrix) | svg |
| 24 | A G T C G A C T C A G T A C T G C T A G T G A C G C T A A T G C G C A T A T C G C G A T A C T G G A T C G T A C G T C A C T G A | NF1(CTF)/LNCAP-NF1-ChIP-Seq(Unpublished)/Homer | 1e-48 | -1.113e+02 | 0.0000 | 368.0 | 7.57% | 1431.5 | 3.22% | motif file (matrix) | svg |
| 25 | A C G T T A C G G A T C A C T G A C G T C T A G A C T G A C T G G A T C C T A G C A T G C T A G | Egr2(Zf)/Thymocytes-Egr2-ChIP-Seq(GSE34254)/Homer | 1e-44 | -1.024e+02 | 0.0000 | 190.0 | 3.91% | 515.6 | 1.16% | motif file (matrix) | svg |
| 26 | T C G A T G A C G T A C C G T A C A G T T G A C A C G T A C T G A G C T A G C T | NeuroG2(bHLH)/Fibroblast-NeuroG2-ChIP-Seq(GSE75910)/Homer | 1e-40 | -9.324e+01 | 0.0000 | 1417.0 | 29.16% | 9330.5 | 21.00% | motif file (matrix) | svg |
| 27 | T C A G A C G T T C G A T A G C A G T C C G T A A C T G G T A C A C G T A C T G A T C G A G T C | Atoh1(bHLH)/Cerebellum-Atoh1-ChIP-Seq(GSE22111)/Homer | 1e-38 | -8.910e+01 | 0.0000 | 954.0 | 19.63% | 5742.9 | 12.92% | motif file (matrix) | svg |
| 28 | T C G A A C G T A C G T C T G A G A T C T C A G G A C T G T C A C G T A A G C T G T C A C T A G A G C T A C G T T C G A | NFIL3(bZIP)/HepG2-NFIL3-ChIP-Seq(Encode)/Homer | 1e-36 | -8.421e+01 | 0.0000 | 843.0 | 17.35% | 4973.7 | 11.19% | motif file (matrix) | svg |
| 29 | T C A G G A C T C A G T C T G A A G C T C T A G G A C T T G C A C T G A A G T C | HLF(bZIP)/HSC-HLF.Flag-ChIP-Seq(GSE69817)/Homer | 1e-36 | -8.405e+01 | 0.0000 | 1102.0 | 22.67% | 6973.1 | 15.69% | motif file (matrix) | svg |
| 30 | C T G A C T G A C A T G A T C G A G C T A T C G G A C T C A T G C T G A G T C A | Tbr1(T-box)/Cortex-Tbr1-ChIP-Seq(GSE71384)/Homer | 1e-35 | -8.260e+01 | 0.0000 | 1431.0 | 29.44% | 9649.4 | 21.71% | motif file (matrix) | svg |
| 31 | T C A G T G A C G T A C C G T A A C G T T G A C A C G T T C A G A G C T G A C T | NeuroD1(bHLH)/Islet-NeuroD1-ChIP-Seq(GSE30298)/Homer | 1e-34 | -7.854e+01 | 0.0000 | 766.0 | 15.76% | 4478.7 | 10.08% | motif file (matrix) | svg |
| 32 | A G C T G T C A C G T A A C G T A C G T C T G A T C A G A T G C | Lhx2(Homeobox)/HFSC-Lhx2-ChIP-Seq(GSE48068)/Homer | 1e-33 | -7.768e+01 | 0.0000 | 1426.0 | 29.34% | 9708.7 | 21.85% | motif file (matrix) | svg |
| 33 | A T G C T A C G A G T C G A C T T G C A C T G A G A C T A C G T C T G A T C A G | LHX9(Homeobox)/Hct116-LHX9.V5-ChIP-Seq(GSE116822)/Homer | 1e-33 | -7.695e+01 | 0.0000 | 1713.0 | 35.25% | 12124.4 | 27.28% | motif file (matrix) | svg |
| 34 | C T G A T G A C T G A C C G T A A C G T T G A C A G C T C T A G A C G T G A C T | Olig2(bHLH)/Neuron-Olig2-ChIP-Seq(GSE30882)/Homer | 1e-31 | -7.168e+01 | 0.0000 | 1790.0 | 36.83% | 12903.3 | 29.04% | motif file (matrix) | svg |
| 35 | T C A G C T A G A T G C G A C T T G C A C G T A A G C T A C G T C T G A T A C G | En1(Homeobox)/SUM149-EN1-ChIP-Seq(GSE120957)/Homer | 1e-29 | -6.783e+01 | 0.0000 | 2084.0 | 42.88% | 15548.2 | 34.99% | motif file (matrix) | svg |
| 36 | A T G C T A C G A G C T T G C A C G T A C G A T A C G T C T G A | DLX5(Homeobox)/BasalGanglia-Dlx5-ChIP-seq(GSE124936)/Homer | 1e-28 | -6.471e+01 | 0.0000 | 1105.0 | 22.74% | 7358.4 | 16.56% | motif file (matrix) | svg |
| 37 | G A C T G C A T C T A G C G A T G A T C T C G A C A T G G A T C | Tgif1(Homeobox)/mES-Tgif1-ChIP-Seq(GSE55404)/Homer | 1e-27 | -6.414e+01 | 0.0000 | 2567.0 | 52.82% | 19959.1 | 44.91% | motif file (matrix) | svg |
| 38 | A T C G A T C G A T G C G A C T T C G A C G T A G C A T A G C T C T G A T A C G | DLX2(Homeobox)/BasalGanglia-Dlx2-ChIP-seq(GSE124936)/Homer | 1e-26 | -6.015e+01 | 0.0000 | 1845.0 | 37.96% | 13669.1 | 30.76% | motif file (matrix) | svg |
| 39 | T C G A C A G T A C T G A G C T C G T A C G T A A C G T A C G T C T G A T A G C | Dlx3(Homeobox)/Kerainocytes-Dlx3-ChIP-Seq(GSE89884)/Homer | 1e-25 | -5.790e+01 | 0.0000 | 978.0 | 20.12% | 6477.7 | 14.58% | motif file (matrix) | svg |
| 40 | G C T A G A C T G A C T T G C A C G T A A G T C C G T A T A G C G A T C G A C T | Eomes(T-box)/H9-Eomes-ChIP-Seq(GSE26097)/Homer | 1e-24 | -5.678e+01 | 0.0000 | 1987.0 | 40.88% | 14999.4 | 33.75% | motif file (matrix) | svg |
| 41 | C G T A C A T G A G T C G A C T T G C A C G T A A C G T A C G T C T G A T C A G | Lhx1(Homeobox)/EmbryoCarcinoma-Lhx1-ChIP-Seq(GSE70957)/Homer | 1e-22 | -5.294e+01 | 0.0000 | 1475.0 | 30.35% | 10693.9 | 24.06% | motif file (matrix) | svg |
| 42 | A T C G A T G C A G T C T A G C G A C T T C G A G C T A G C A T A G C T C T G A | DLX1(Homeobox)/BasalGanglia-Dlx1-ChIP-seq(GSE124936)/Homer | 1e-21 | -4.928e+01 | 0.0000 | 1620.0 | 33.33% | 12030.6 | 27.07% | motif file (matrix) | svg |
| 43 | A G C T C A T G G C A T G A T C T G C A C T A G G A T C A C G T | Tgif2(Homeobox)/mES-Tgif2-ChIP-Seq(GSE55404)/Homer | 1e-21 | -4.925e+01 | 0.0000 | 2599.0 | 53.48% | 20703.3 | 46.59% | motif file (matrix) | svg |
| 44 | C T A G T C G A T G A C A G T C C G T A A C T G G T A C A C G T A C T G A C T G | BHLHA15(bHLH)/NIH3T3-BHLHB8.HA-ChIP-Seq(GSE119782)/Homer | 1e-21 | -4.910e+01 | 0.0000 | 1163.0 | 23.93% | 8180.8 | 18.41% | motif file (matrix) | svg |
| 45 | A G C T A C G T A C T G A T G C A G T C C G T A C T G A T A C G | NF1-halfsite(CTF)/LNCaP-NF1-ChIP-Seq(Unpublished)/Homer | 1e-21 | -4.850e+01 | 0.0000 | 1265.0 | 26.03% | 9043.7 | 20.35% | motif file (matrix) | svg |
| 46 | C T G A T C A G G T A C G C T A A C T G T G A C G C A T C A T G | SCL(bHLH)/HPC7-Scl-ChIP-Seq(GSE13511)/Homer | 1e-20 | -4.811e+01 | 0.0000 | 2990.0 | 61.52% | 24345.3 | 54.78% | motif file (matrix) | svg |
| 47 | C T G A T C A G C A G T C T A G A C T G C T A G G A T C A T C G A C T G C T G A T C A G G A T C | Sp5(Zf)/mES-Sp5.Flag-ChIP-Seq(GSE72989)/Homer | 1e-20 | -4.715e+01 | 0.0000 | 485.0 | 9.98% | 2855.1 | 6.42% | motif file (matrix) | svg |
| 48 | A G T C C G A T A C T G A T C G T G A C G C T A C A T G A T C G T G A C C G A T A C T G T A G C G T A C G T C A | Tlx?(NR)/NPC-H3K4me1-ChIP-Seq(GSE16256)/Homer | 1e-20 | -4.695e+01 | 0.0000 | 348.0 | 7.16% | 1867.9 | 4.20% | motif file (matrix) | svg |
| 49 | A G T C A C G T A C T G A G C T A C G T A C G T G T C A A G T C | Foxo1(Forkhead)/RAW-Foxo1-ChIP-Seq(Fan\_et\_al.)/Homer | 1e-19 | -4.391e+01 | 0.0000 | 1716.0 | 35.31% | 13019.4 | 29.30% | motif file (matrix) | svg |
| 50 | G A C T C A G T A G C T C G A T A G T C G A T C A G T C C G T A A T G C T C A G | Rbpj1(?)/Panc1-Rbpj1-ChIP-Seq(GSE47459)/Homer | 1e-16 | -3.709e+01 | 0.0000 | 1176.0 | 24.20% | 8610.9 | 19.38% | motif file (matrix) | svg |
| 51 | C T G A C T A G A C T G G C A T C T A G G C A T A T C G C T G A C G T A G T C A | Tbx21(T-box)/GM12878-TBX21-ChIP-Seq(Encode)/Homer | 1e-15 | -3.641e+01 | 0.0000 | 922.0 | 18.97% | 6516.9 | 14.67% | motif file (matrix) | svg |
| 52 | C T G A C T A G A T C G A G C T A C T G G A C T A G T C C T G A | Tbx5(T-box)/HL1-Tbx5.biotin-ChIP-Seq(GSE21529)/Homer | 1e-15 | -3.640e+01 | 0.0000 | 2262.0 | 46.54% | 18104.2 | 40.74% | motif file (matrix) | svg |
| 53 | C G T A C G T A A G C T G A C T T G C A G T C A A C G T A G C T C T G A T C A G | Lhx3(Homeobox)/Neuron-Lhx3-ChIP-Seq(GSE31456)/Homer | 1e-15 | -3.556e+01 | 0.0000 | 1924.0 | 39.59% | 15123.3 | 34.03% | motif file (matrix) | svg |
| 54 | C T A G C T G A T C A G A T C G A G C T C A T G G A C T A G T C C T G A T G C A | Tbx6(T-box)/ESC-Tbx6-ChIP-Seq(GSE93524)/Homer | 1e-15 | -3.505e+01 | 0.0000 | 864.0 | 17.78% | 6076.3 | 13.67% | motif file (matrix) | svg |
| 55 | C G T A C T A G C A T G A G C T A C T G C G A T A T C G C G T A G T C A G T C A | Tbet(T-box)/CD8-Tbet-ChIP-Seq(GSE33802)/Homer | 1e-15 | -3.480e+01 | 0.0000 | 982.0 | 20.21% | 7055.6 | 15.88% | motif file (matrix) | svg |
| 56 | T G A C A G T C C G T A A C T G G T A C A C G T A C T G A C G T G A C T G A T C | Twist2(bHLH)/Myoblast-Twist2.Ty1-ChIP-Seq(GSE127998)/Homer | 1e-14 | -3.345e+01 | 0.0000 | 1405.0 | 28.91% | 10672.9 | 24.02% | motif file (matrix) | svg |
| 57 | A C T G C A G T A C G T C G T A C G T A A C G T A C T G C T G A | Nkx6.1(Homeobox)/Islet-Nkx6.1-ChIP-Seq(GSE40975)/Homer | 1e-13 | -3.175e+01 | 0.0000 | 2699.0 | 55.53% | 22258.4 | 50.09% | motif file (matrix) | svg |
| 58 | C G T A C T G A C T A G C G T A C G T A A G T C C G T A C A G T G C A T G T C A C G A T A C T G A C G T G C A T G A T C | PGR(NR)/EndoStromal-PGR-ChIP-Seq(GSE69539)/Homer | 1e-13 | -3.093e+01 | 0.0000 | 309.0 | 6.36% | 1806.7 | 4.07% | motif file (matrix) | svg |
| 59 | T A C G T G C A A G T C C G T A A C G T T G A C A C G T A C T G A C T G G C A T | TCF4(bHLH)/SHSY5Y-TCF4-ChIP-Seq(GSE96915)/Homer | 1e-13 | -3.071e+01 | 0.0000 | 1239.0 | 25.49% | 9343.1 | 21.02% | motif file (matrix) | svg |
| 60 | A G T C G C A T C G T A C G T A G T A C A C G T A C T G G A T C G A T C T C G A | BMYB(HTH)/Hela-BMYB-ChIP-Seq(GSE27030)/Homer | 1e-11 | -2.692e+01 | 0.0000 | 1294.0 | 26.63% | 9947.3 | 22.38% | motif file (matrix) | svg |
| 61 | C G T A C G T A C G T A G C A T G C A T T A C G G T A C G A C T A C T G C G T A A T C G A C G T G T A C C G T A A G C T | Bach1(bZIP)/K562-Bach1-ChIP-Seq(GSE31477)/Homer | 1e-11 | -2.603e+01 | 0.0000 | 84.0 | 1.73% | 329.3 | 0.74% | motif file (matrix) | svg |
| 62 | T C A G T A C G T A G C A C G T A C T G C G A T A G T C C G T A T A C G A G T C | Meis1(Homeobox)/MastCells-Meis1-ChIP-Seq(GSE48085)/Homer | 1e-11 | -2.581e+01 | 0.0000 | 1267.0 | 26.07% | 9756.7 | 21.96% | motif file (matrix) | svg |
| 63 | G A C T G T A C T G C A A C G T G A T C G C T A T C G A A C G T A G T C C G T A | Pdx1(Homeobox)/Islet-Pdx1-ChIP-Seq(SRA008281)/Homer | 1e-10 | -2.489e+01 | 0.0000 | 1043.0 | 21.46% | 7875.3 | 17.72% | motif file (matrix) | svg |
| 64 | T G A C C T G A C T A G C T G A C G T A A G T C C T G A A C G T G C A T T A G C G C A T A T C G G A C T G A C T G A T C | GRE(NR),IR3/RAW264.7-GRE-ChIP-Seq(Unpublished)/Homer | 1e-10 | -2.471e+01 | 0.0000 | 249.0 | 5.12% | 1462.7 | 3.29% | motif file (matrix) | svg |
| 65 | T A G C G C A T A G T C G A T C A T G C G A C T C T A G A C T G A C T G C T G A A C T G C T A G A G T C T G A C C G A T | GLIS3(Zf)/Thyroid-Glis3.GFP-ChIP-Seq(GSE103297)/Homer | 1e-10 | -2.458e+01 | 0.0000 | 653.0 | 13.44% | 4632.2 | 10.42% | motif file (matrix) | svg |
| 66 | C T A G A G T C T A C G T A C G T G A C C G T A A C T G T A G C G C A T C A T G A T G C A G C T | Ascl1(bHLH)/NeuralTubes-Ascl1-ChIP-Seq(GSE55840)/Homer | 1e-10 | -2.456e+01 | 0.0000 | 982.0 | 20.21% | 7370.7 | 16.59% | motif file (matrix) | svg |
| 67 | G A C T C T A G C T A G A G T C T G C A A C T G A C G T A C G T C T A G T C A G | AMYB(HTH)/Testes-AMYB-ChIP-Seq(GSE44588)/Homer | 1e-9 | -2.163e+01 | 0.0000 | 1236.0 | 25.43% | 9652.2 | 21.72% | motif file (matrix) | svg |
| 68 | C G A T C T A G A C G T G T C A C G T A C G T A A G T C C G T A | Foxo3(Forkhead)/U2OS-Foxo3-ChIP-Seq(E-MTAB-2701)/Homer | 1e-9 | -2.075e+01 | 0.0000 | 856.0 | 17.61% | 6439.2 | 14.49% | motif file (matrix) | svg |
| 69 | G A C T C T A G G A T C C A G T A C T G C T G A A T G C G C A T A T G C C T G A | MafA(bZIP)/Islet-MafA-ChIP-Seq(GSE30298)/Homer | 1e-9 | -2.074e+01 | 0.0000 | 659.0 | 13.56% | 4794.2 | 10.79% | motif file (matrix) | svg |
| 70 | G T C A G C A T A C T G G T A C G A C T A C T G G C T A A T C G C A G T G T A C C G T A A G C T | Nrf2(bZIP)/Lymphoblast-Nrf2-ChIP-Seq(GSE37589)/Homer | 1e-8 | -1.961e+01 | 0.0000 | 69.0 | 1.42% | 285.6 | 0.64% | motif file (matrix) | svg |
| 71 | C G T A C G A T G A C T G A C T T G A C C T G A A T G C C T G A T A G C A G T C A C G T T C G A C A T G T A C G G A C T A T C G G A C T A C G T C T G A T C G A C G T A | Brachyury(T-box)/Mesoendoderm-Brachyury-ChIP-exo(GSE54963)/Homer | 1e-8 | -1.944e+01 | 0.0000 | 284.0 | 5.84% | 1818.9 | 4.09% | motif file (matrix) | svg |
| 72 | A C T G A C G T C A T G A T C G A T C G T G A C A C T G A T C G A T C G T G C A C T G A C G T A | E2F3(E2F)/MEF-E2F3-ChIP-Seq(GSE71376)/Homer | 1e-8 | -1.898e+01 | 0.0000 | 206.0 | 4.24% | 1237.6 | 2.78% | motif file (matrix) | svg |
| 73 | T C A G A G C T A C G T A C G T G T A C G A T C C G T A C T A G C A T G G T C A C G T A T C G A | STAT4(Stat)/CD4-Stat4-ChIP-Seq(GSE22104)/Homer | 1e-8 | -1.894e+01 | 0.0000 | 875.0 | 18.00% | 6666.3 | 15.00% | motif file (matrix) | svg |
| 74 | A G C T T G A C C G A T C G A T C T A G A C G T C A G T C A G T G C T A A G T C | FOXK1(Forkhead)/HEK293-FOXK1-ChIP-Seq(GSE51673)/Homer | 1e-7 | -1.793e+01 | 0.0000 | 1151.0 | 23.68% | 9074.8 | 20.42% | motif file (matrix) | svg |
| 75 | A T G C T C G A T A C G A C G T A T G C A G T C A C G T A G T C A G T C G A T C | Znf263(Zf)/K562-Znf263-ChIP-Seq(GSE31477)/Homer | 1e-7 | -1.718e+01 | 0.0000 | 991.0 | 20.39% | 7727.0 | 17.39% | motif file (matrix) | svg |
| 76 | T A C G T C G A C A G T A C T G G C T A A T G C C G A T G T A C C G T A A C T G T A G C C G T A | NF-E2(bZIP)/K562-NFE2-ChIP-Seq(GSE31477)/Homer | 1e-7 | -1.710e+01 | 0.0000 | 74.0 | 1.52% | 336.4 | 0.76% | motif file (matrix) | svg |
| 77 | C T A G C T A G A G T C T C A G A C T G A C G T A C G T C T G A | MYB(HTH)/ERMYB-Myb-ChIPSeq(GSE22095)/Homer | 1e-7 | -1.709e+01 | 0.0000 | 1406.0 | 28.93% | 11337.2 | 25.51% | motif file (matrix) | svg |
| 78 | C G A T T G C A T G C A G A T C C G T A A C T G T G A C G A C T C A T G A C T G | Tcf21(bHLH)/ArterySmoothMuscle-Tcf21-ChIP-Seq(GSE61369)/Homer | 1e-7 | -1.703e+01 | 0.0000 | 630.0 | 12.96% | 4674.0 | 10.52% | motif file (matrix) | svg |
| 79 | C T A G A C T G C T A G T C A G T C A G T A C G C T A G A C T G | Maz(Zf)/HepG2-Maz-ChIP-Seq(GSE31477)/Homer | 1e-7 | -1.695e+01 | 0.0000 | 561.0 | 11.54% | 4104.7 | 9.24% | motif file (matrix) | svg |
| 80 | A T G C G A C T A C G T C T A G A C G T A C G T A C G T C T G A G A T C G C T A A G C T C G T A | Foxa2(Forkhead)/Liver-Foxa2-ChIP-Seq(GSE25694)/Homer | 1e-6 | -1.604e+01 | 0.0000 | 871.0 | 17.92% | 6747.2 | 15.18% | motif file (matrix) | svg |
| 81 | T C A G A T C G G A C T A C T G G A C T C A G T C T A G C G T A G T A C C G T A C T A G A T C G | Tbx20(T-box)/Heart-Tbx20-ChIP-Seq(GSE29636)/Homer | 1e-6 | -1.601e+01 | 0.0000 | 180.0 | 3.70% | 1094.8 | 2.46% | motif file (matrix) | svg |
| 82 | G C A T G C A T C T G A A C G T C T G A A C G T C G T A C G T A C G T A A G T C G T C A G T C A | Foxf1(Forkhead)/Lung-Foxf1-ChIP-Seq(GSE77951)/Homer | 1e-6 | -1.550e+01 | 0.0000 | 1200.0 | 24.69% | 9613.2 | 21.63% | motif file (matrix) | svg |
| 83 | T G C A A G C T A C G T C T A G G A T C C T A G G A T C G T C A C T G A A G T C | CEBP(bZIP)/ThioMac-CEBPb-ChIP-Seq(GSE21512)/Homer | 1e-6 | -1.534e+01 | 0.0000 | 649.0 | 13.35% | 4893.9 | 11.01% | motif file (matrix) | svg |
| 84 | A G T C C G A T C T G A C G T A A C G T C A G T T C A G T G A C | Isl1(Homeobox)/Neuron-Isl1-ChIP-Seq(GSE31456)/Homer | 1e-6 | -1.522e+01 | 0.0000 | 1836.0 | 37.78% | 15250.6 | 34.32% | motif file (matrix) | svg |
| 85 | G A C T C A G T G A T C G A T C A C G T G A T C C T G A T A C G C G T A G T C A | STAT6(Stat)/Macrophage-Stat6-ChIP-Seq(GSE38377)/Homer | 1e-6 | -1.456e+01 | 0.0000 | 534.0 | 10.99% | 3960.4 | 8.91% | motif file (matrix) | svg |
| 86 | C T G A T C A G A G T C C G T A A T C G A T G C C G A T A C T G A G T C G A C T A T C G A G T C | MyoD(bHLH)/Myotube-MyoD-ChIP-Seq(GSE21614)/Homer | 1e-6 | -1.396e+01 | 0.0000 | 463.0 | 9.53% | 3392.9 | 7.64% | motif file (matrix) | svg |
| 87 | C T G A C G A T C T A G C G T A A G C T C G A T C A G T C T G A G A C T C T A G C T A G A T G C | PBX2(Homeobox)/K562-PBX2-ChIP-Seq(Encode)/Homer | 1e-5 | -1.281e+01 | 0.0000 | 878.0 | 18.07% | 6951.8 | 15.64% | motif file (matrix) | svg |
| 88 | T A C G C T G A C T A G T C G A C G T A A G T C C T G A A T C G G C A T T A G C G A C T A C T G A C G T A G C T A G T C G A C T | GRE(NR),IR3/A549-GR-ChIP-Seq(GSE32465)/Homer | 1e-5 | -1.211e+01 | 0.0000 | 129.0 | 2.65% | 781.8 | 1.76% | motif file (matrix) | svg |
| 89 | A T G C T C G A A G T C A G C T A C G T G T A C A G T C G C T A C T A G C A T G G T C A C T G A T C A G A G T C | Stat3+il21(Stat)/CD4-Stat3-ChIP-Seq(GSE19198)/Homer | 1e-5 | -1.156e+01 | 0.0000 | 555.0 | 11.42% | 4248.5 | 9.56% | motif file (matrix) | svg |
| 90 | A T G C C T G A A T C G T A C G A G T C C G A T T C A G C G A T C T A G A G C T G T C A G T C A C G T A A G T C C G T A T A C G C T G A | Fox:Ebox(Forkhead,bHLH)/Panc1-Foxa2-ChIP-Seq(GSE47459)/Homer | 1e-4 | -1.133e+01 | 0.0001 | 1002.0 | 20.62% | 8102.9 | 18.23% | motif file (matrix) | svg |
| 91 | T A C G T A G C G C T A C G A T C T A G A C G T C A G T C A G T G C T A A G T C G T C A G C A T | FOXK2(Forkhead)/U2OS-FOXK2-ChIP-Seq(E-MTAB-2204)/Homer | 1e-4 | -1.095e+01 | 0.0001 | 629.0 | 12.94% | 4902.9 | 11.03% | motif file (matrix) | svg |
| 92 | G A C T C T A G A T G C A G T C G T C A T A C G A T G C A T C G | HIC1(Zf)/Treg-ZBTB29-ChIP-Seq(GSE99889)/Homer | 1e-4 | -1.082e+01 | 0.0001 | 1193.0 | 24.55% | 9805.0 | 22.06% | motif file (matrix) | svg |
| 93 | A G T C C T A G C T A G A G T C G A T C G T A C A G T C C T A G A G T C A G T C A G T C G T A C | Sp2(Zf)/HEK293-Sp2.eGFP-ChIP-Seq(Encode)/Homer | 1e-4 | -1.073e+01 | 0.0001 | 579.0 | 11.91% | 4486.2 | 10.10% | motif file (matrix) | svg |
| 94 | A T G C A G T C G C A T A G C T A C G T T C A G C G A T A G C T G A T C A T C G | Sox10(HMG)/SciaticNerve-Sox3-ChIP-Seq(GSE35132)/Homer | 1e-4 | -1.069e+01 | 0.0001 | 1406.0 | 28.93% | 11696.3 | 26.32% | motif file (matrix) | svg |
| 95 | T A C G A T G C G A C T A C T G A G C T A G T C G T C A T G C A A C G T A G T C G C T A T G C A | Pknox1(Homeobox)/ES-Prep1-ChIP-Seq(GSE63282)/Homer | 1e-4 | -1.058e+01 | 0.0001 | 166.0 | 3.42% | 1092.5 | 2.46% | motif file (matrix) | svg |
| 96 | G T A C G C A T C T G A C G T A G A C T A G C T C A T G T G C A C T G A A C G T G A C T C G T A | Prop1(Homeobox)/GHFT1-PROP1.biotin-ChIP-Seq(GSE77302)/Homer | 1e-4 | -1.051e+01 | 0.0001 | 810.0 | 16.67% | 6478.6 | 14.58% | motif file (matrix) | svg |
| 97 | G A T C G C A T C T G A C G T A A G C T A G C T C G T A T C G A C T G A A C G T G C A T C G T A | Phox2a(Homeobox)/Neuron-Phox2a-ChIP-Seq(GSE31456)/Homer | 1e-4 | -1.016e+01 | 0.0002 | 510.0 | 10.49% | 3926.3 | 8.84% | motif file (matrix) | svg |
| 98 | G A C T C G T A A C G T A C T G A G T C C G T A C T G A C G T A C A G T A C G T G T C A T C A G | Brn1(POU,Homeobox)/NPC-Brn1-ChIP-Seq(GSE35496)/Homer | 1e-4 | -9.786e+00 | 0.0003 | 377.0 | 7.76% | 2824.9 | 6.36% | motif file (matrix) | svg |
| 99 | G T C A T G C A G C T A A G T C C G T A A C T G T G A C G C A T T C A G C A G T | Ap4(bHLH)/AML-Tfap4-ChIP-Seq(GSE45738)/Homer | 1e-4 | -9.482e+00 | 0.0003 | 785.0 | 16.15% | 6316.1 | 14.21% | motif file (matrix) | svg |
| 100 | T A G C T A G C G A C T C T A G A G C T A G T C G T C A T G C A A C G T A T G C G C T A T G C A | Pbx3(Homeobox)/GM12878-PBX3-ChIP-Seq(GSE32465)/Homer | 1e-4 | -9.411e+00 | 0.0004 | 179.0 | 3.68% | 1222.0 | 2.75% | motif file (matrix) | svg |
| 101 | A G C T A G T C A G T C A C G T C T A G A C G T A C G T A C G T C G T A A G T C G A T C C G T A | FOXP1(Forkhead)/H9-FOXP1-ChIP-Seq(GSE31006)/Homer | 1e-3 | -9.185e+00 | 0.0004 | 485.0 | 9.98% | 3756.2 | 8.45% | motif file (matrix) | svg |
| 102 | T C A G T G A C G T A C T G C A G T A C C T A G G T A C A T G C A G T C G T C A A G T C G A C T | Klf9(Zf)/GBM-Klf9-ChIP-Seq(GSE62211)/Homer | 1e-3 | -9.173e+00 | 0.0004 | 178.0 | 3.66% | 1219.3 | 2.74% | motif file (matrix) | svg |
| 103 | T A C G C T G A T C G A C G A T C T A G C T A G T C G A C T G A T C G A T C G A C G T A T C G A G C A T C A T G C G T A T A C G G C A T T G A C C G T A A G C T | NFAT:AP1(RHD,bZIP)/Jurkat-NFATC1-ChIP-Seq(Jolma\_et\_al.)/Homer | 1e-3 | -9.142e+00 | 0.0005 | 165.0 | 3.40% | 1117.2 | 2.51% | motif file (matrix) | svg |
| 104 | C T A G T A G C A G T C G T C A C T G A A C G T C G T A C G T A C G T A G C T A | Hoxd13(Homeobox)/ChickenMSG-Hoxd13.Flag-ChIP-Seq(GSE86088)/Homer | 1e-3 | -8.995e+00 | 0.0005 | 1779.0 | 36.60% | 15149.9 | 34.09% | motif file (matrix) | svg |
| 105 | A T G C G T A C C G T A A G C T G C A T T A C G A G C T A G C T A G T C A G C T | Sox6(HMG)/Myotubes-Sox6-ChIP-Seq(GSE32627)/Homer | 1e-3 | -8.810e+00 | 0.0006 | 1480.0 | 30.45% | 12484.3 | 28.09% | motif file (matrix) | svg |
| 106 | G A C T C G A T C T G A G T C A G A C T C G A T T C G A C G T A G C T A G C T A T G A C G T A C C G T A A C T G T G C A C G A T A C T G A C G T | Pitx1:Ebox(Homeobox,bHLH)/Hindlimb-Pitx1-ChIP-Seq(GSE41591)/Homer | 1e-3 | -8.685e+00 | 0.0007 | 219.0 | 4.51% | 1561.4 | 3.51% | motif file (matrix) | svg |
| 107 | A C G T T C G A T C G A A G T C G T C A T A C G A T G C A C G T A C T G A G C T | Myf5(bHLH)/GM-Myf5-ChIP-Seq(GSE24852)/Homer | 1e-3 | -8.548e+00 | 0.0008 | 430.0 | 8.85% | 3318.6 | 7.47% | motif file (matrix) | svg |
| 108 | A G T C T A G C G A C T A C G T C T A G A C G T A C G T A C G T C T G A A G T C G C T A G A C T C G T A C T A G A C T G | Foxa3(Forkhead)/Liver-Foxa3-ChIP-Seq(GSE77670)/Homer | 1e-3 | -8.165e+00 | 0.0012 | 367.0 | 7.55% | 2803.1 | 6.31% | motif file (matrix) | svg |
| 109 | C G T A G C T A C G A T C T A G A C G T G T C A C G T A C G T A A G T C C G T A T G C A T A C G | FoxL2(Forkhead)/Ovary-FoxL2-ChIP-Seq(GSE60858)/Homer | 1e-3 | -7.780e+00 | 0.0017 | 1042.0 | 21.44% | 8670.8 | 19.51% | motif file (matrix) | svg |
| 110 | C G T A G A C T C G A T A T C G G T A C G C A T C A T G C G T A T A C G G C A T G T A C C G T A C A T G A T G C G C T A C T A G G C A T G C A T G C A T G A C T | MafB(bZIP)/BMM-Mafb-ChIP-Seq(GSE75722)/Homer | 1e-3 | -7.653e+00 | 0.0019 | 391.0 | 8.05% | 3028.0 | 6.81% | motif file (matrix) | svg |
| 111 | T A G C G T A C C G T A C T A G A C T G T G C A C G T A A T G C C G T A A T C G | AR-halfsite(NR)/LNCaP-AR-ChIP-Seq(GSE27824)/Homer | 1e-3 | -7.427e+00 | 0.0024 | 2657.0 | 54.67% | 23259.5 | 52.34% | motif file (matrix) | svg |
| 112 | C T G A A G C T A C G T A C G T A G T C G A C T G A C T C T G A C T G A C T A G C G T A C G T A | STAT6(Stat)/CD4-Stat6-ChIP-Seq(GSE22104)/Homer | 1e-3 | -7.267e+00 | 0.0027 | 504.0 | 10.37% | 4009.2 | 9.02% | motif file (matrix) | svg |
| 113 | C G T A C G T A C G T A G C A T G C A T A C T G G T A C G A C T C T A G G C T A T A C G G A C T T G A C C G T A A G C T | NFE2L2(bZIP)/HepG2-NFE2L2-ChIP-Seq(Encode)/Homer | 1e-3 | -7.004e+00 | 0.0035 | 85.0 | 1.75% | 542.0 | 1.22% | motif file (matrix) | svg |
| 114 | T A G C G A T C G T A C C T G A G C A T C T G A C G T A T G C A C G T A G A T C | Hoxa13(Homeobox)/ChickenMSG-Hoxa13.Flag-ChIP-Seq(GSE86088)/Homer | 1e-3 | -6.925e+00 | 0.0038 | 2561.0 | 52.70% | 22426.0 | 50.47% | motif file (matrix) | svg |
| 115 | T C G A A G T C C G T A A T C G A T G C C G A T A C T G A G T C A G C T A C T G | Tcf12(bHLH)/GM12878-Tcf12-ChIP-Seq(GSE32465)/Homer | 1e-3 | -6.925e+00 | 0.0038 | 527.0 | 10.84% | 4225.6 | 9.51% | motif file (matrix) | svg |
| 116 | C T A G A T G C A T G C C G A T A C T G G A C T A T G C G C T A T G A C A G C T T A G C G C T A | PBX1(Homeobox)/MCF7-PBX1-ChIP-Seq(GSE28007)/Homer | 1e-2 | -6.842e+00 | 0.0041 | 62.0 | 1.28% | 373.0 | 0.84% | motif file (matrix) | svg |
| 117 | G T C A A C G T C T A G G T C A C G T A G C A T C G T A C G T A G C A T C A G T A G T C C G T A C A G T C T A G C T G A | OCT:OCT(POU,Homeobox,IR1)/NPC-Brn2-ChIP-Seq(GSE35496)/Homer | 1e-2 | -6.828e+00 | 0.0041 | 26.0 | 0.53% | 120.8 | 0.27% | motif file (matrix) | svg |
| 118 | G A C T C T A G C T A G C T A G A C T G T C G A C T G A C T A G C T A G C T A G G T A C G T C A | ZNF467(Zf)/HEK293-ZNF467.GFP-ChIP-Seq(GSE58341)/Homer | 1e-2 | -6.805e+00 | 0.0041 | 433.0 | 8.91% | 3424.1 | 7.71% | motif file (matrix) | svg |
| 119 | C T A G T A C G G A T C G T A C G C T A A G C T A G C T G T C A T C G A T A G C | Nanog(Homeobox)/mES-Nanog-ChIP-Seq(GSE11724)/Homer | 1e-2 | -6.747e+00 | 0.0043 | 3168.0 | 65.19% | 28030.7 | 63.08% | motif file (matrix) | svg |
| 120 | C G T A T A G C A G T C C T A G C A G T C T A G C T G A G T A C G C A T T C G A C G T A G C A T A G C T C T A G T C G A | PAX3:FKHR-fusion(Paired,Homeobox)/Rh4-PAX3:FKHR-ChIP-Seq(GSE19063)/Homer | 1e-2 | -6.714e+00 | 0.0045 | 223.0 | 4.59% | 1657.2 | 3.73% | motif file (matrix) | svg |
| 121 | A T C G T G A C A T G C C T G A T C A G G A C T A G T C C G A T T C A G T C G A C A T G C T A G C T A G C G T A C T A G C T A G C T G A C T A G C T A G A T G C | ZSCAN22(Zf)/HEK293-ZSCAN22.GFP-ChIP-Seq(GSE58341)/Homer | 1e-2 | -6.661e+00 | 0.0047 | 41.0 | 0.84% | 223.4 | 0.50% | motif file (matrix) | svg |
| 122 | C T A G T C G A C G A T C T A G G C A T C A G T C T A G G A T C C G T A G T C A | CEBP:AP1(bZIP)/ThioMac-CEBPb-ChIP-Seq(GSE21512)/Homer | 1e-2 | -6.628e+00 | 0.0048 | 661.0 | 13.60% | 5404.7 | 12.16% | motif file (matrix) | svg |
| 123 | G C T A A G C T T A C G G T C A C T G A C G A T C T G A G C A T C A G T A G T C | Brn2(POU,Homeobox)/NPC-Brn2-ChIP-Seq(GSE35496)/Homer | 1e-2 | -6.346e+00 | 0.0063 | 122.0 | 2.51% | 846.2 | 1.90% | motif file (matrix) | svg |
| 124 | T A C G T A C G G T A C A T C G A C T G T A C G T C G A C T G A T C G A A T C G | E2F6(E2F)/Hela-E2F6-ChIP-Seq(GSE31477)/Homer | 1e-2 | -6.118e+00 | 0.0078 | 143.0 | 2.94% | 1022.0 | 2.30% | motif file (matrix) | svg |
| 125 | T G C A A G C T C A T G C G T A A G C T A C T G G A T C G T C A C G T A A G C T | Atf4(bZIP)/MEF-Atf4-ChIP-Seq(GSE35681)/Homer | 1e-2 | -6.113e+00 | 0.0078 | 241.0 | 4.96% | 1829.6 | 4.12% | motif file (matrix) | svg |
| 126 | T C A G T A G C G A C T C A T G C T G A A T C G G C A T G T A C C G T A A C T G T A G C T G C A | MafK(bZIP)/C2C12-MafK-ChIP-Seq(GSE36030)/Homer | 1e-2 | -5.944e+00 | 0.0092 | 200.0 | 4.12% | 1494.8 | 3.36% | motif file (matrix) | svg |
| 127 | A G T C A T C G G C A T C A T G A C T G A T C G C G A T C T A G A C T G A G C T T G A C G A C T | Gli2(Zf)/GM2-Gli2-ChIP-Chip(GSE112702)/Homer | 1e-2 | -5.716e+00 | 0.0114 | 135.0 | 2.78% | 968.1 | 2.18% | motif file (matrix) | svg |
| 128 | A T G C G A T C C G A T A C G T A C G T A C T G C A G T A G C T | Sox3(HMG)/NPC-Sox3-ChIP-Seq(GSE33059)/Homer | 1e-2 | -5.690e+00 | 0.0116 | 1489.0 | 30.64% | 12824.4 | 28.86% | motif file (matrix) | svg |
| 129 | C G T A A C G T A G C T C G A T C T A G G T A C C G T A A G C T C G T A G C T A | Oct4(POU,Homeobox)/mES-Oct4-ChIP-Seq(GSE11431)/Homer | 1e-2 | -5.635e+00 | 0.0122 | 539.0 | 11.09% | 4404.2 | 9.91% | motif file (matrix) | svg |
| 130 | T C G A T A G C G T C A A C T G C T A G C G T A C G A T A C T G A C G T A C T G A C T G A C G T | ETS:RUNX(ETS,Runt)/Jurkat-RUNX1-ChIP-Seq(GSE17954)/Homer | 1e-2 | -5.590e+00 | 0.0126 | 49.0 | 1.01% | 296.6 | 0.67% | motif file (matrix) | svg |
| 131 | T A C G T C A G G A T C G T A C T C G A G A C T G C T A G C T A G C T A C G T A G A T C G T C A | CDX4(Homeobox)/ZebrafishEmbryos-Cdx4.Myc-ChIP-Seq(GSE48254)/Homer | 1e-2 | -5.407e+00 | 0.0151 | 984.0 | 20.25% | 8337.4 | 18.76% | motif file (matrix) | svg |
| 132 | C T G A C T A G T C G A C G T A A T G C C G T A A T C G C G A T T A G C G C A T A T C G G C A T A G C T G A T C G A C T A G C T | ARE(NR)/LNCAP-AR-ChIP-Seq(GSE27824)/Homer | 1e-2 | -5.230e+00 | 0.0178 | 238.0 | 4.90% | 1840.1 | 4.14% | motif file (matrix) | svg |
| 133 | C T A G C T A G A C T G A G C T G A C T A G C T A C G T C T A G G T C A C T G A A C T G T C A G C T G A A C G T C T A G G C T A C T G A A C G T C G T A C T A G A C T G C G T A A C T G A G C T A C G T | ZFP3(Zf)/HEK293-ZFP3.GFP-ChIP-Seq(GSE58341)/Homer | 1e-2 | -5.192e+00 | 0.0184 | 2.0 | 0.04% | 1.6 | 0.00% | motif file (matrix) | svg |
| 134 | G T A C C G T A C G T A T A C G G C A T G T A C C G T A C A T G A G T C C G T A C G T A C G A T G C A T G C A T G A C T | MafF(bZIP)/HepG2-MafF-ChIP-Seq(GSE31477)/Homer | 1e-2 | -5.161e+00 | 0.0188 | 288.0 | 5.93% | 2267.4 | 5.10% | motif file (matrix) | svg |
| 135 | C T G A A C G T A C G T A C G T A G T C G A C T C G A T C T G A A C T G C G T A C G T A T C G A | STAT5(Stat)/mCD4+-Stat5-ChIP-Seq(GSE12346)/Homer | 1e-2 | -5.159e+00 | 0.0188 | 303.0 | 6.23% | 2395.9 | 5.39% | motif file (matrix) | svg |
| 136 | A G T C G T A C A G C T C T A G A G T C C G A T A C T G C G T A A C T G G T C A | Zic(Zf)/Cerebellum-ZIC1.2-ChIP-Seq(GSE60731)/Homer | 1e-2 | -4.819e+00 | 0.0261 | 503.0 | 10.35% | 4143.2 | 9.32% | motif file (matrix) | svg |
| 137 | T G A C C G A T A C T G A C T G A C T G G A C T A C T G A C G T A C T G A C T G G A T C G A T C | EKLF(Zf)/Erythrocyte-Klf1-ChIP-Seq(GSE20478)/Homer | 1e-2 | -4.791e+00 | 0.0267 | 114.0 | 2.35% | 825.2 | 1.86% | motif file (matrix) | svg |
| 138 | T A G C T C A G C A T G G C A T A G C T C G A T A T G C C G T A C G T A G T C A | CHR(?)/Hela-CellCycle-Expression/Homer | 1e-2 | -4.660e+00 | 0.0302 | 651.0 | 13.40% | 5452.1 | 12.27% | motif file (matrix) | svg |
